# Hold your breath!

**DOI:** 10.7554/eLife.12523

**Published:** 2015-12-16

**Authors:** Katharina Lust, Joachim Wittbrodt

**Affiliations:** Centre for Organismal Studies Heidelberg, Ruprecht-Karls-University Heidelberg, Heidelberg, Germany; Centre for Organismal Studies Heidelberg, Ruprecht-Karls-University Heidelberg, Heidelberg, Germanyjochen.wittbrodt@cos.uni-heidelberg.de

**Keywords:** newt, regeneration, neural stem cells, neurogenesis, reactive oxygen species, Other

## Abstract

Reactive oxygen species produced in response to changes in the level of oxygen in water can promote the regeneration of brain tissue in newts.

**Related research article** Hameed LS, Berg DA, Belnoue L, Jensen LD, Cao Y, Simon A. 2015. Environmental changes in oxygen tension reveal ROS-dependent neurogenesis and regeneration in the adult newt brain. *eLife* **4**:e08422. doi: 10.7554/eLife.08422

**Image:** A drop in the oxygen level of water can kill brain cells in newts.

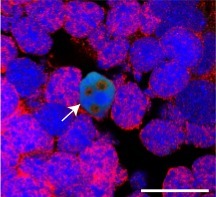

Regeneration, the process by which an animal restores lost cells or body parts, occurs throughout the animal kingdom, including vertebrates, planarians and cnidarians such as hydra (Tanaka & Reddien, 2011). However, the ability to regenerate varies between species, and within a species it can be different for different body parts. Salamanders, such as axolotls and newts, possess the most impressive regenerative capabilities among the vertebrates, and are capable of regenerating entire organs and limbs.

One salamander in particular, the red spotted newt, has the ability to do something remarkable – to regenerate brain cells and tissue in response to damage caused by chemicals (Parish et al., 2007; Berg et al., 2010). They are also able to cope with low levels of oxygen (hypoxia) in the water they live in. Since hypoxia and re-oxygenation can cause tissue damage, András Simon of the Karolinska Institute and colleagues – including Shahul Hameed as first author – decided to investigate the effects of these two processes on cell death and regeneration in the forebrain of the newt (Hameed et al., 2015).

Previous studies have shown that the death of cells after an injury leads to the activation of the immune system and an inflammatory response. Immune cells called microglia start to divide (proliferate) and migrate to the site of the injury, where they engulf and destroy the dead neurons (Wake et al., 2013). Hameed et al. found that the newts were able to survive in the hypoxic conditions for days. However, exposing the newts to hypoxia followed by re-oxygenation caused some neurons in the forebrain to die, and also led to an increase in the number of active microglia cells in the brain. They also found that cells called ependymoglia stem cells – which replace neurons as part of normal body maintenance and after injury (Parish et al., 2007; Berg et al., 2010) – replaced the neurons that died after hypoxia and re-oxygenation (Figure 1).

**Figure 1.**
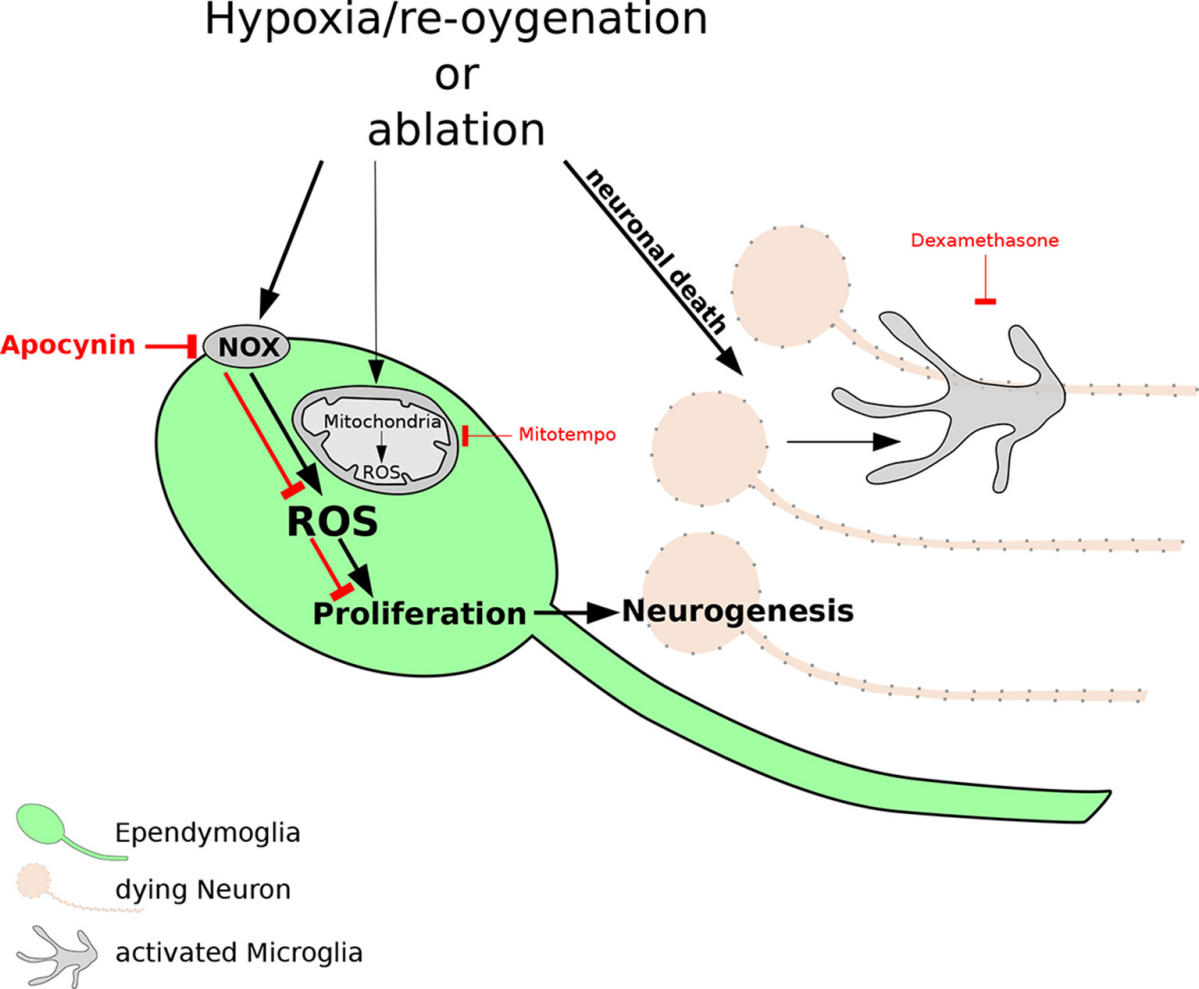
The reactive oxygen species (ROS) that are produced after damage to neurons are necessary for regeneration of the newt brain. Exposure to hypoxia followed by re-oxygenation, or deliberate chemical damage (ablation), leads to the loss of neurons (orange) in red spotted newts, and also causes immune cells called microglia (grey) to proliferate. In ependymoglia stem cells (green), ROS are produced inside mitochondria and by NOX proteins at the cell membrane. Only NOX-derived ROS, which can be inhibited by the drug apocynin (bold red lines), are necessary for the proliferation of ependymoglia stem cells and the subsequent regeneration of lost neurons. Inhibiting the microglia with a drug called Dexamethasone (thin red lines) has no effect on the proliferation of the ependymoglia stem cells, which suggests that inflammation does not influence the regeneration of neurons. Bold black lines represent processes that are necessary for regeneration; the processes represented by thin black lines are not necessary for regeneration.

Periods of hypoxia followed by re-oxygenation are known to induce 'oxidative' stress in cells and tissues: this can lead to the production of reactive oxygen species (ROS) that often damage proteins and other molecules, and it can also destabilize the genome (Schieber & Chandel, 2014). However, it has recently become clear that ROS can also promote cell division (Le Belle et al., 2011) and can even be necessary for regeneration (Love et al., 2013). Hameed et al. found that the production of ROS increased in ependymoglia stem cells after hypoxia and re-oxygenation. The ROS were produced by the mitochondria in the cells, and by NADPH oxidase complex (NOX) proteins in the cell membrane. Experiments with drugs that inhibit the production of ROS showed that only the ROS that were produced by the NOX proteins are required for the ependymoglia stem cells to increase in number.

Recently it was shown that inflammation can promote regeneration of the zebrafish brain (Kyritsis et al., 2012) and axolotl limbs (Godwin et al., 2013). However, Hameed et al. found that the proliferation of ependymoglia stem cells in the newt brain does not depend on inflammation. Additionally, increases in ROS did not influence the activation of microglia. Therefore, unlike in many other systems, there is not a close relationship between hypoxia and inflammation in the newt brain.

Finally, Hameed et al. addressed the role of ROS in the regeneration of dopaminergic neurons in the midbrain under normal oxygen conditions. Chemical damage to these neurons led to increased production of ROS in the ependymoglia stem cells, and the experiments confirmed that this increased production was required for the neurons to regenerate. However, the high levels of ROS observed would normally kill neurons and other cells, which raises the question: how can ependymoglia stem cells cope with these levels of ROS and use them as a cue to start dividing?

Another question raised by these findings is whether ROS play a similar role in brain regeneration in other animals. Previous studies have shown that brain regeneration in zebrafish depends on inflammation. However, since zebrafish are also able to survive in environments that can become hypoxic, it is possible that they may also have another mechanism for brain regeneration that involves ROS. How different regenerative abilities are either lost or gained during evolution has been discussed for a long time, but is not well understood and is extremely difficult to study. It will be exciting to observe how the recent findings by Hameed et al. will stimulate and contribute to these studies.
